# Food Choice Architecture: An Intervention in a Secondary School and its Impact on Students’ Plant-based Food Choices

**DOI:** 10.3390/nu7064426

**Published:** 2015-06-02

**Authors:** Hannah Ensaff, Matt Homer, Pinki Sahota, Debbie Braybrook, Susan Coan, Helen McLeod

**Affiliations:** 1 Nutrition & Dietetics, School of Health & Wellbeing, Leeds Beckett University, Leeds, LS1 3HE, UK; E-Mails: p.sahota@leedsbeckett.ac.uk (P.S.); d.braybrook@leedsbeckett.ac.uk (D.B.); s.coan@leedsbeckett.ac.uk (S.C.); 2 School of Education, University of Leeds, Leeds, LS2 9JT, UK; E-Mail: m.s.homer@education.leeds.ac.uk; 3 Health & Wellbeing Service, Leeds City Council, Leeds, LS12 1DB, UK; E-Mail: helen.mcleod@leeds.gov.uk

**Keywords:** food choice, adolescents, school nutrition, choice architecture, intervention

## Abstract

With growing evidence for the positive health outcomes associated with a plant-based diet, the study’s purpose was to examine the potential of shifting adolescents’ food choices towards plant-based foods. Using a real world setting of a school canteen, a set of small changes to the choice architecture was designed and deployed in a secondary school in Yorkshire, England. Focussing on designated food items (whole fruit, fruit salad, vegetarian daily specials, and sandwiches containing salad) the changes were implemented for six weeks. Data collected on students’ food choice (218,796 transactions) enabled students’ (980 students) selections to be examined. Students’ food choice was compared for three periods: baseline (29 weeks); intervention (six weeks); and post-intervention (three weeks). Selection of designated food items significantly increased during the intervention and post-intervention periods, compared to baseline (baseline, 1.4%; intervention 3.0%; post-intervention, 2.2%) χ^2^(2) = 68.1, *p* < 0.001. Logistic regression modelling also revealed the independent effect of the intervention, with students 2.5 times as likely (*p* < 0.001) to select the designated food items during the intervention period, compared to baseline. The study’s results point to the influence of choice architecture within secondary school settings, and its potential role in improving adolescents’ daily food choices.

## 1. Introduction

Adolescents’ diet in the UK is high in saturated fat and sugar, along with low fruit and vegetable consumption [1]; generally marked by poor food choice, a propensity for fast or grab-and-go foods has been illustrated in previous studies [2,3,4]. These choices, typified by low intake of plant-based foods, are mirrored in school canteens, where students commonly bypass freshly prepared nutrient rich meals [3].

Choice architecture, how a choice is presented and its influence on subsequent decision-making behaviour, is well-established [5,6,7]. Food choice architecture specifically encompasses all aspects of how a food choice is framed, and how this influences the food selections actually made, e.g., the relative availability and presentation of different foods. Within public health, there is growing impetus behind libertarian paternalism, where choice architecture is manipulated to ‘nudge’ behaviour, without removing the freedom to choose [5,7]. Internationally, governments are exploring nudge theory and its exploitation over the imposition of government legislation. This is particularly the case, where strong armed regulation is not perceived as the answer, for example in food choice where lifestyle factors clearly impact on a nation’s health [8].

There is therefore a growing literature on the role of choice architecture in promoting better food choice, and nutrition interventions are increasingly emphasising the role of behavioural nudge tactics in shifting people’s behaviour towards better decisions in terms of health and wellbeing. Some studies have shown how adjustments to the accessibility and presentation of ‘healthy’ foods can promote their selection and consumption [9,10,11,12,13]. Other studies have demonstrated the value of initiatives where foods are accorded attractive names [14], or mentioned in point of purchase prompts [15]. Nevertheless, inconsistencies have been reported in the effectiveness of choice architecture in changing food choice behaviour, and there is a need for further research in the area [6,7,16].

The school dining environment provides a sound setting for such research, both in terms of instigating changes to the choice architecture and determining their effectiveness. Food provision within secondary schools in England typically consists of a wide range of foods in a cafeteria-style setting. Freshly prepared daily specials, e.g., beef lasagne or vegetable curry, constitute a three or four week menu cycle, which is complemented by more ‘grab-and-go’ options, e.g., pizza and sandwiches, as well as ‘sweet’ food items, e.g., cookies, fruit.

Eating behaviour is largely automatic, reliant on non-cognitive processing [17] and heavily influenced by the environment and contextual food cues. School canteens in UK secondary schools are often time-pressured environments—rendering food choice even more susceptible to automatic decision-making, and consequently environmental nudge strategies. Adjusting the choice architecture with reference to these factors could be a means of nudging adolescents towards healthier food decisions.

This study sought to evaluate nudge strategies in a real world setting of a school dining environment. In particular, the study looked to test whether a *set* of complementary changes to the choice architecture could effectively shift a school population’s food choice towards more plant-based foods.

## 2. Experimental Section

This study was approved by Leeds Beckett University’s Faculty of Health and Social Sciences’ Research Ethics Committee. All data were anonymised and used in strict adherence to the UK Data Protection Act.

Two secondary schools (1 intervention and 1 control school) within a large city in Yorkshire were recruited. Both were inner city schools with raised levels of deprivation indicated by high Free School Meal (FSM) profiles (the Free School Meal programme is a national programme providing a free school meal for students of low income families) (*intervention school*: 33%; *control school*: 42%; national average in England: 14.6% [18]). The percentage of students with English not as a first language (ENFL) was also substantially higher than the national average (*intervention school*: 26%; *control school*: 39%; national average in England (14.3%) [18]). Both schools were of a similar size with approximately eleven hundred students (age 11–18 years) on roll and utilized the same national catering provider. The schools’ kitchens operated the same three-week menu cycle of freshly prepared meals, which incorporated two daily specials—one of which was vegetarian (e.g., lentil casserole, vegetable lasagne). In addition to these, ‘grab-and-go’ options were available every day; these included pizza, pasta, jacket potatoes, salads, sandwiches and baguettes of various fillings. ‘Sweet’ food items were also provided daily, e.g., cookies, traybakes, hot puddings, fruit, and fruit pots.

Both schools used a cashless system, whereby catering staff keyed in students’ selections on a touchscreen till. In this way, the point of sale data automatically captured every selection made by a student, in the form of a transaction, comprising a food item code, alongside the time and date of purchase. Each food item code varied in terms of how specific it was; some codes referred to only one item (e.g., cheese and tomato pizza), whilst other codes were slightly more general (e.g., value sandwich, deluxe baguette). In total there were 112 and 92 different codes in use at the intervention and control schools, respectively.

### 2.1. Intervention

Of all the items already on offer to students, specific foods of high plant-based content were designated as the targets for the intervention. These foods were chosen on the basis of their ingredients (using menus and recipes provided by the catering company), as well as the specificity of their codes as employed by the cashless system—since the point of sale data would be used to monitor their selection. The designated food items were: freshly prepared vegetarian daily specials; sandwiches containing salad; fruit pot (fruit salad in a disposable plastic pot); and whole fruit. Two additional item codes were introduced in order to capture data relating to the already existing vegetarian daily specials and sandwiches containing salad. Previously, these items were not distinguished at the till, and instead were keyed in as daily specials (alongside other non-vegetarian daily specials) and sandwiches (a generic code), respectively.

**Figure 1:**
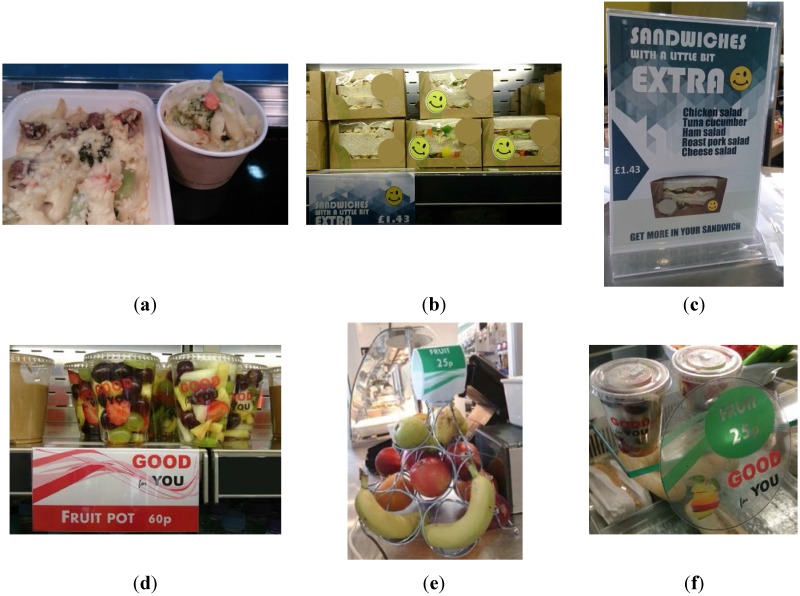
Some of the nudge strategies implemented for designated food items during the intervention: (**a**) disposable pots for freshly prepared vegetarian daily specials; (**b**) stickers on sandwiches containing salad; (**c**) poster promoting sandwiches containing salad; (**d**) stickers and end of shelf labels for fruit pots; (**e**) pyramid display stand holding whole fruit; and (**f**) window sticker promoting whole fruit.

The intervention consisted of a set of small changes to the choice architecture (nudge strategies) targeting the designated plant-based foods. Initially, visits were conducted at both schools, during which full service in the canteen was observed. Using notes and photographs taken during the observation visits, the canteen layout was sketched on a scale plan, to appreciate how students made their selections of different foods as they navigated the canteen. This, as well as a review of previous studies conducted in the field, contributed to an initial list of potential nudge strategies. Following close consultation and discussion with the catering staff, catering company and school leadership, the design of the intervention was finalised. The components to the intervention included preferential product placement and arrangement, labelling, and presentation, as outlined in Table 1 and Figure 1. Importantly, the intervention did not involve any changes to the food on offer to students, and the changes were not publicised overtly to students.

**Table 1:** Overview of the nudge strategies implemented for designated food items.

**Freshly prepared vegetarian daily specials**	
Disposable pots/trays	Disposable pots/trays were used to serve meals (a change from a dinner plate)
Prefilled pots/trays	Meals displayed in prefilled pots/trays, ready for selection
Poster displayed in holder	Daily posters promoting the freshness of the meal: “Today’s SPECIAL—Make a fresh choice” and featuring a photograph of the meal, a descriptive name and the price
Window sticker in display unit	Window stickers promoting the freshness of the meal: “Today’s SPECIAL—Make a fresh choice” and the price
**Sandwiches containing salad**	
Stickers on sandwich packaging	Stickers with smiley faces (a different face for each of the six weeks of the intervention)
End of shelf label	Weekly label promoting “Sandwiches with a little bit extra”, and showing the relevant smiley face and the price
Poster displayed in holder	Weekly poster promoting “Sandwiches with a little bit extra”, “Get more in your sandwich”, and showing the relevant smiley face, relevant sandwiches and the price
**Fruit pots**	
Stickers on fruit pots	Transparent stickers “GOOD for YOU”
End of shelf label	Label promoting fruit pots with “GOOD for YOU” and the price
Window sticker in display unit	Window stickers promoting fruit pots with “GOOD for YOU” and the price
**Whole fruit**	
Pyramid display stand	Stand holding individual pieces of fruit, with the price
Prominent position	Repositioned near till
Window sticker in display unit	Window stickers promoting fruit with “GOOD for YOU” and the price
*All promoted food items had increased numbers on display*	

The intervention was implemented during the academic year 2013–2014 for six weeks in the summer term. Students made choices within the adjusted choice architecture for the six weeks of the intervention, before it was reinstated to its baseline state for the remainder of the summer term.

Data were collected from both schools for the duration of the academic year 2013–2014 (Y1), and the previous year 2012–2013 (Y0). This amounted to a period of 188 school days (Y1) and 190 school days (Y0) for the intervention school, and a period of 189 school days (Y1) and 188 school days (Y0) for the control school.

### 2.2. Statistical Analysis

The data collected were imported and analysed using SPSS statistical software (IBM SPSS Statistics for Windows, Version 21, IBM Corp. Armonk, NY, USA). The data were cleaned and those transactions relating to students only were extracted to form new datasets for analysis. These comprised more than 200,000 transactions (*intervention school*: 218,796 transactions in Y1; 222,015 transactions in Y0; *control school*: 236,557 transactions in Y1; 242,991 transactions in Y0), and incorporated the school food choice of all students utilising the school canteen at any point during the respective year (*intervention school*, Y1, 980 students; Y0, 1132 students)—not all students used the canteen, e.g., some brought in a packed lunch every day. For the control school, unique identifiers were not issued to all students (due to the particular cashless system employed) and so, whilst student transactions were tracked, the numbers of students using the school canteen was not available.

Each food item available in the canteens was assigned to a broad and narrow category, based on the food type, detailed caterer’s food descriptions and the observation visits. The broad categories were ‘main’ foods, savoury snacks, ‘sweet’ foods, beverages, and accompaniments. Each of these broad categories was then divided into narrow categories, e.g., ‘sweet’ foods were categorised as cookies/traybakes, hot puddings, fruit, *etc*. All food items with a fruit, vegetable or salad (FVS) component were also coded within the datasets, in order to provide a proxy indicator of plant-based food selection. Students’ selections in Y1, 2013–2014 were considered for three time periods: baseline (29-week period); intervention (6-week period); and post-intervention (3-week period). During the previous year Y0, 2012–2013, the equivalent weeks were considered.

Initially, overall food choice patterns at both schools and for both years were examined using chi-square tests. For both schools, comparisons were made in order to establish how different the patterns of food choice were between Y1 (2013–2014) and Y0 (2012–2013) (between year), and also how different the patterns of food choice were between baseline, intervention and post-intervention for each of the two years (within year).

The popularity of food items at the intervention school was then considered using chi-square tests. The number of items selected by students as a percentage of all items selected within the relevant categories was evaluated, e.g., students’ selections of fruit within the broad category of ‘sweet’ foods, or students’ selections of vegetarian daily specials within the broad category of ‘main’ foods. For the vegetarian daily specials and sandwiches containing salad, baseline data were limited to three weeks only, as these relied on two new codes specifically introduced for this study. Effect size, phi, is used to assess the strength of differences in the chi-square tests.

Finally, logistic regression models were conducted for the intervention school to see if item selection (or not) could be predicted by the intervention period, with covariates of student FSM eligibility, student year group, the day of the week and item price.

## 3. Results

The intervention was completed for the proposed duration, and with all components implemented as planned. Intervention fidelity was assessed through close liaison with the school, including a series of observation visits during the intervention period, as well as interim data analysis.

Overall food choice patterns at both schools and for both years were examined using chi-square tests. Students’ overall selection at the intervention school during the intervention was significantly different to the rest of the year and the previous year. When comparing the three time periods (baseline, intervention and post-intervention) in Y1 (2013–2014) to the equivalent weeks in Y0 (2012–2013), the pattern of food selection was more different for the intervention and post-intervention periods at the intervention school—reflected in the accompanying effect sizes, which were moderate and progressively larger during the intervention and post-intervention periods compared to baseline (baseline φ = 0.37; intervention φ = 0.44; post-intervention φ = 0.46). For the control school, the patterns of food selection produced more comparable effect sizes between respective periods in the two years and overall these were more modest (baseline φ = 0.39; intervention φ = 0.39; post intervention φ = 0.40). When considering within year differences, the pattern of food selection at the intervention school was significantly different for the intervention period. The difference was more pronounced for the intervention period during Y1 than Y0 for the intervention school, again reflected in the effect sizes measured (Y1, φ = 0.32; Y0, φ = 0.26), compared to the control school, which showed smaller differences between Y1 and Y0 (Y1, φ = 0.23; Y0, φ = 0.21).

**Figure 2:**
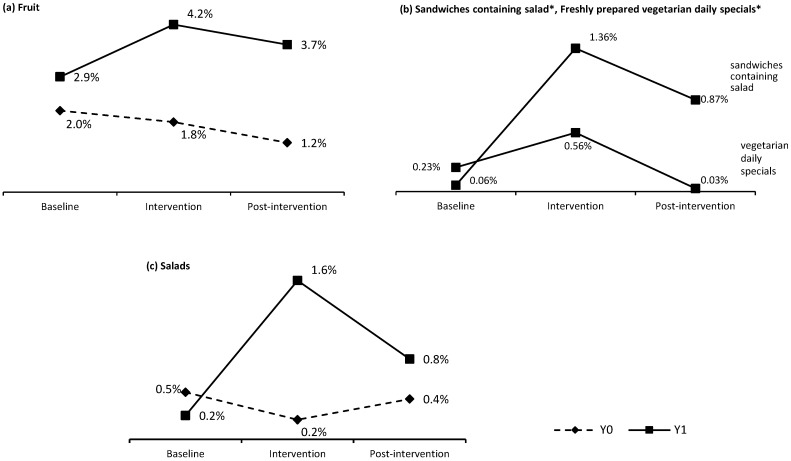
Selection of foods for baseline, intervention and post-intervention periods of Y1 (*n* = 980) and the equivalent weeks during the previous year Y0 (*n* = 1132), (number of items selected as a percentage of the relevant broad category): (**a**) fruit items (Y1:53,835; Y0:50,148 ‘sweet’ food items); (**b**) sandwiches containing salad, freshly prepared vegetarian daily specials (Y1:18,972 ‘main’ food items); and (**c**) salad items (Y1:76,168; Y0 86,849 ‘main’ food items). * baseline data limited to three weeks.

Students’ selections for Y1 and the equivalent weeks in Y0 at the intervention school are presented in Figure 2. The effect sizes for all presented data were relatively small (reflecting the generally low percentage uptake at baseline). However, they were consistently larger during the intervention year (Y1) than the previous year (Y0). At the intervention school, selection of fruit items (fruit pots and whole fruit) increased significantly during the intervention and post-intervention periods compared to baseline (baseline 2.9%; intervention 4.2%; post-intervention 3.7%) χ^2^(2) = 44.32, *p* < 0.001, φ = 0.03. This was largely attributed to the increased selection of fruit pots during the intervention from 0.8% to 1.9%. Selection of the vegetarian daily specials increased from 0.2% of ‘main’ foods to 0.6% during the intervention χ^2^(2) = 23.41, *p* < 0.001, φ = 0.04. Likewise, selection of sandwiches containing salad increased from 0.06% to 1.36% χ^2^(2) = 64.75, *p* < 0.001, φ = 0.06. When considering all food items promoted during the intervention, selection increased significantly during the intervention and post-intervention periods compared to baseline (baseline 1.4%; intervention 3.0%; post-intervention 2.2%) χ^2^(2) = 68.07, *p* < 0.001, φ = 0.04. Interestingly, selection of salad items (which were not a designated food for the intervention) also significantly increased during the intervention (baseline: 0.2%; intervention: 1.6%; post-intervention: 0.8%) χ^2^(2) = 379.57, *p* < 0.001, φ = 0.07. When considering all FVS food items, their selection by students increased during the intervention year (baseline 1.5%; intervention 3.8%; post-intervention 2.6%) χ^2^(2) = 118.64, *p* < 0.001, φ = 0.06.

Logistic regression analysis revealed the independent effect of the intervention on the uptake of promoted food items. Table 2 presents the odds ratios for the selection of food items during the intervention and post-intervention periods (relative to baseline). Controlling for student year group, free school meal entitlement (a measure of socioeconomic status), day of the week and price, students were 2.5 times as likely (*p* < 0.001) to select the designated items during the intervention period, than at baseline. As well as the independent effect of the intervention on the selection of promoted food items, an effect on the selection of FVS items was observed, and students were three times as likely to choose a fruit, vegetable or salad item during the intervention (relative to baseline). The spillover effect seen in students’ selection of salad items (small or large boxed salads, or side salads) is also evident in the logistic regression results with students 7.5 times as likely to select salad items during the intervention compared to baseline.

**Table 2:** Overall independent effect of time (intervention and post-intervention relative to baseline) on students’ selection of food items.

Food	*n*	Intervention				Post-intervention			Nagelkerke *R*^2^
		OR	95% CI	*p*		OR	95% CI	*p*	
Vegetarian daily specials *	18,725	2.94	1.51–5.69	0.001		0.53	0.06–4.33	0.551	0.43
Sandwiches containing salad *	18,725	35.57	8.79–144.01	<0.001		26.00	6.15–109.90	<0.001	0.08
Fruit pots	53,471	2.41	1.98–2.93	<0.001		2.51	1.85–3.42	<0.001	0.16
Whole fruit **	53,471	1.15	0.99–1.35	0.074		1.02	0.77–1.35	0.890	0.02
Promoted foods *	34,320	2.49	2.03–3.06	<0.001		2.18	1.68–2.82	<0.001	0.06
Salad	75,048	7.53	5.94–9.54	<0.001		5.80	3.73–9.02	<0.001	0.18
FVS *	34,320	3.04	2.50–3.69	<0.001		2.48	1.94–3.16	<0.001	0.06

Adjusted for year group, FSM eligibility, day of the week and price. All selections are relative to baseline and considered within foods’ relevant broad categories. Abbreviations: OR, Odds Ratio; 95% CI: 95% Confidence Interval; FVS: Fruit, Vegetable or Salad items. * Short baseline used in the analysis. ** Selection adjusted for year group, FSM eligibility, day of the week but not price as price didn’t vary within whole fruit.

## 4. Discussion

This study evaluated a choice architecture intervention within a secondary school dining environment, and to the authors’ knowledge this is the first study of its type within the UK. Small adjustments to the choice architecture nudged students towards more plant-based food choices. Overall, students were 2.5 times as likely to choose a designated promoted item. This study’s findings concur with other research [9,11,12,13], where small adjustments to food items’ accessibility, availability and/or presentation altered their subsequent selection. Nudge strategies previously explored include changing the positioning, display and presentation of foods [9,11,12], using colour coded labelling [13], descriptive names [11,14] and verbal prompting [15], as well as implementing a convenience lunch line for the selection of ‘healthy’ foods [10,11]. The potential of nudge strategies in driving school food choices, as evidenced by this current study, corresponds with previous research conducted in schools in the US [10,11,14,15]. In this intervention, plant-based foods were designated and targeted; previous choice architecture interventions in schools have focussed on fruit [11,15], vegetables [11,14] and ‘healthier’ foods [10]. Whilst it is important to be clear about the rationale and strategy with respect to selecting the foods to be promoted, well designed and executed nudge strategies can avail themselves to many target foods. Furthermore, the value of a set of complementary nudge strategies in shifting a population’s food choice behaviour has been seen in this study; this concurs with previous work where a combination of nudges has been applied [11].

The study also revealed selection of salad items increased from 0.2% to 1.6% during the intervention, with students more than seven times as likely to choose salad during the intervention compared to before. This spillover effect is worthy of further inquiry. Whilst the potential of unintended consequences to changing choice architecture have been previously described [19]—it is particularly encouraging that this is a positive unintended outcome. It also contributed to the overall change observed in students’ selection of FVS items during the intervention. In this way the choice architecture was successfully manipulated to shift food choice towards more plant-based foods, and students were three times as likely to choose fruit, vegetable or salad items during the intervention.

The data from the cashless system provided genuine insights into students’ food choice, and enabled the impact of the intervention to be accurately evaluated. The power and value of using such data to examine students’ dietary patterns has been shown previously [3,20], and this study adds to the literature, in particular with respect to monitoring a change accompanying a nutrition intervention.

Interventions such as these, aimed at creating environments to promote better decisions, are valuable. Policy makers are exploring the role of choice architecture in facilitating healthier decisions, as an alternative to government legislation. This study extends current literature on choice architecture and the use of nudge strategies to encourage more favourable food decisions, in particular within a school food environment.

### 4.1. Strengths and Limitations

In considering this study’s findings, the relevant strengths and limitations should be acknowledged. This study used the food choices over two academic years for two school populations. The analysis’ strength comes from the extent and duration of the data collected. Data generated from cashless systems provide an accurate and long-term account of food choices compared to self-reported dietary data. Further, the inclusion of a control school, as well as the preceding year’s data served as valuable comparators in the study. In particular, seasonal changes in food selection were accounted for by the use of the equivalent weeks in the previous year. The national catering company employed at both schools had practices and arrangements common to many secondary schools in England, indicating that the findings of this study may be widely applicable.

There were differences in the specificity of the coding of the foods; whilst some food items were very specific and related to one item only, other codes were more generic. All data presented, however, relate to well-defined broad and narrow categories or food codes. The data analysed is point of sale data and not consumption data, and this should be recognised. For the purposes of this study, however, the impact on selection was key, and so the use of this data was particularly appropriate.

Foods designated as targets for the intervention were selected on the basis of constituent ingredients, as well as the relevant food item codes. More specific coding, e.g., where every individual product had its own dedicated code, would have provided a greater level of detail to the testing of the nudge strategies, e.g., the impact on each of the different sandwiches containing salad, rather than the collective sandwiches containing salad. This was, however constrained by a limited number of codes available on the point of sale systems.

Inconsistencies in catering staff keying in students’ selections at the till should be acknowledged. This is particularly relevant to the vegetarian daily specials and the sandwiches containing salad as they related to new food item codes introduced specifically for this study. The staff was briefed on this change and the requirement to use the new codes, however this potential source of error should be recognised. Likewise, analyses involving these promoted foods were only possible for the year of the intervention and using a shortened baseline of three weeks.

Statistical analysis has ignored dependency in the data, *i.e.*, that the same students were involved throughout. This will have the effect of p-value estimates being a little smaller than they should be – hence our emphasis on effect sizes, although the large size of the data implies that most if not all statistical results are highly significant (*i.e.*, *p* < 0.001).

The schools in this study were characterised by higher than average FSM and ENFL profiles. Any consideration of this study’s results should be within the context of the population sample.

### 4.2. Implications for Policy and Practice

This study provides encouraging evidence for the effect of small changes on students’ selection in a school canteen. Opportunities to improve students’ diet at lunchtime raises the potential for substantial impact on students’ wellbeing and performance, and the findings have implications for practice and policy.

In implementing nudge strategies, there is a range of opportunities, e.g., product placement, accessibility, and labelling. Indeed, this study has shown how a *set* of changes can be used effectively—and to a large extent the changes made were determined by the food items promoted and the existing layout of the canteen. Regardless of the type or combination of changes, however, engagement with the catering staff is a key determinant of successful implementation. It is also critical that any changes are not onerous and do not detract from the day-to-day service of the kitchen. This is all itself contingent on early discussion and involvement with the catering team.

Whilst significant changes were observed during the intervention, it is interesting to note the low starting point for the designated food items. So, for example during the academic year 2013–2014 the average daily uptake of fruit was three fruit pots and six pieces of fruit—compared to 236 cookies and traybakes per day. Thus, whilst the nudges employed in this intervention have a substantial and valuable contribution to make, further strategies (involving choice architecture and otherwise) are needed to improve adolescents’ food choice in schools, particularly in the longer term.

This study incorporated changes to the promoted foods; changes to other foods deemed less favourable should also be considered, e.g., making these items less prominent and accessible by repositioning them.

Areas for further research have also been highlighted by this study. Whilst the findings illustrate how a set of small changes can nudge food choice towards more favourable options, the relative contributions of the different nudge strategies are not explicit. It may be possible to attribute increases in food selection to specific changes to those foods; this relationship, however, may not be so straightforward. Further work to tease out the relative influences is required. This is especially the case when considering, for example, the spillover effect observed in salads (not a designated food for the intervention, and not a focus of any of the nudge strategies).

Whilst the higher rates of selection were not maintained post-intervention, these were still elevated compared to baseline. There is a need for further research to explore the relevant factors involved in maintaining a sustained response. In particular, the requirement or otherwise for continuous changes to the choice architecture is an interesting question.

## 5. Conclusions

Increasingly, schools are seen as a proponent of student health and wellbeing. This study’s findings evidence the role of choice architecture in improving adolescents’ dietary behaviour within the school dining environment. By making simple changes to the food choice architecture, students’ selections were shifted towards more favourable options. In the pursuit of improving adolescents’ food choice, school catering practice and school food policies should reflect the place of choice architecture within schools.
